# Systematic review of gut microbiota composition, metabolic alterations, and the effects of treatments on PCOS and gut microbiota across human and animal studies

**DOI:** 10.3389/fmicb.2025.1549499

**Published:** 2025-05-14

**Authors:** Aya Hanna, Hassan Abbas, Fayez Yassine, Alia AlBush, Melhem Bilen

**Affiliations:** ^1^Department of Experimental Pathology, Immunology and Microbiology, Faculty of Medicine, American University of Beirut, Beirut, Lebanon; ^2^Centre for Infectious Diseases Research, American University of Beirut, Beirut, Lebanon; ^3^World Health Organization (WHO) Collaborating Centre for Reference and Research on Bacterial Pathogens, Beirut, Lebanon

**Keywords:** polycystic ovary syndrome, gastrointestinal microbiome, endocrine system diseases, fecal microbiota transplantation, metagenomics, metabolomics

## Abstract

**Introduction:**

Polycystic ovary syndrome (PCOS) is an endocrine disorder affecting around 12% of women globally, associated with infertility and various comorbidities. Emerging evidence suggests a crucial role of gut microbiota in PCOS pathophysiology, prompting research to investigate alterations in gut microbial composition in patients with PCOS.

**Methods:**

This systematic review aims to analyze human and animal studies that compare gut microbiota composition, gut-derived metabolites, and treatment interventions in PCOS patients versus healthy controls. A comprehensive literature search was conducted using PubMed, Scopus, and Web of Science, yielding studies examining gut microbiota, metabolomic shifts, and treatment responses in PCOS models and human populations.

**Results:**

Our analysis revealed decreases in alpha diversity in PCOS patients, with more pronounced changes in beta diversity in animal models. Specific bacterial taxa, such as *Bacteroides vulgatus, Escherichia-Shigella* and *Lactobacillus*, showed implication in PCOS pathogenesis, suggesting potential microbial markers. Furthermore, discrepancies between human and animal studies show the need for humanized mouse models to bridge this gap. Interventions like probiotics and fecal microbiota transplantation (FMT) showed varying levels of efficacy, with FMT emerging as a more promising but invasive option, offering live bacteriotherapy as a potential therapeutic alternative. Alterations in gut-derived metabolites, including short-chain fatty acids and bile acids, highlighted the multifaceted nature of PCOS, with implications extending to metabolic, hormonal, and gut-brain axis disruptions.

**Discussion:**

In conclusion, PCOS exhibits complex interactions between gut microbiota and metabolic pathways, necessitating further research with standardized methods and larger sample sizes to elucidate the microbiome's role in PCOS.

## Graphical Abstract

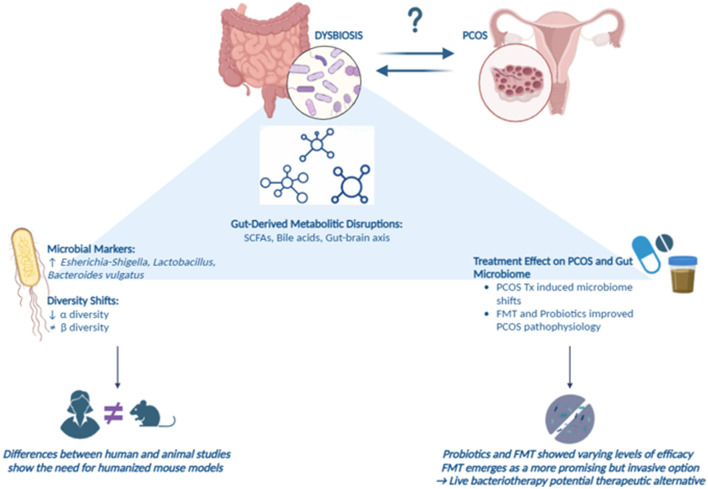

## 1 Introduction

Polycystic ovary syndrome (PCOS) is an endocrine disorder impacting 11–13% of women worldwide, posing substantial health and economic challenges (Salari et al., 2024; The Lancet Regional Health-Europe, 2022). Characterized by hyperandrogenism, irregular menstruation, and polycystic ovaries (Teede et al., 2023), PCOS is the most common cause of infertility and is associated with increased risks of pregnancy complications (Dennett and Simon, 2015). But despite its high prevalence, PCOS diagnosis can be elusive, coupled with limited treatment options due to an incomplete understanding of its underlying mechanisms (Gibson-Helm et al., 2017). Furthermore, PCOS is linked to a wide range of comorbidities, including obesity, hyperandrogenism, metabolic dysfunction, infertility, obstetric complications, psychological disorders, cancer, and long-term postmenopausal morbidity and mortality risks (Stener-Victorin et al., 2024).

Emerging research has begun to explore the link between PCOS and the gut microbiome, recognizing the potential role of gut microbial dysbiosis in disease pathogenesis (Min et al., 2023). The gut microbiota, dominated by the bacterial phyla *Firmicutes* and *Bacteroidetes*, interacts with the host through various neural, endocrine, and immune pathways, affecting not only gut health but also systemic diseases (Fava et al., 2019; Daniel et al., 2021). These microbial communities are influenced by factors such as diet, lifestyle, and age, making them dynamic targets for intervention (Hou et al., 2022). Alterations in gut microbiota have been implicated in several metabolic and inflammatory disorders, raising the possibility that they may also play a role in the development and progression of PCOS (Afzaal et al., 2022).

While initial studies have suggested distinct gut microbiota profiles in women with PCOS, the evidence remains inconsistent, and the precise relationship between gut dysbiosis and PCOS pathogenesis is unclear. To address this, we conducted a systematic review to analyze gut microbiota composition, metabolomic changes, and the impact of treatments on PCOS and gut microbiota across human and animal studies. Our aim is to provide reliable evidence of an association between PCOS and the microbiome and to facilitate the translation of findings from animal models to human applications.

## 2 Material and methods

### 2.1 Search strategy

We conducted a systematic search of PubMed, Scopus, and Web of Science (WOS) from start to 28/06/2024 for all original articles comparing the gastrointestinal microbiotas of patients with PCOS to those of controls, including studies linking PCOS with gut microbiota treatments, gut microbial metabolites, or gut microbial composition in both human and animal-based research. For PubMed, our search strategy involved a combination of MeSH terms and keywords related to the gastrointestinal microbiota (e.g., “Gastrointestinal Microbiome,” “Gut Microbiota,” “Intestinal Flora”) and PCOS (e.g., “Polycystic Ovary Syndrome,” “Stein-Leventhal Syndrome,” “Sclerocystic Ovaries”). Our detailed search queries for PubMed and other databases are included in Supplementary material 1. Articles were exported to EndNote, where duplicates were removed using the EndNote function, followed by manual curation to ensure accuracy.

### 2.2 Inclusion and exclusion criteria

The inclusion criteria were studies involving human subjects diagnosed with PCOS, relevant animal models (mice and rats), clinical trials, or observational studies exploring gut microbiome composition, metabolites, and treatment interventions. Studies had to compare PCOS groups with healthy controls, assess differences before and after treatment, or compare treatment versus placebo groups.

Exclusion criteria included studies that did not focus primarily on PCOS and the gut microbiome, studies that lacked a control group for comparison, and studies investigating other microbiomes (e.g., blood, skin, or oral microbiomes). Additionally, studies not published in English, review articles or commentaries, and those with insufficient data for analysis were excluded. To ensure accuracy and minimize bias, two independent authors reviewed study eligibility based on the inclusion and exclusion criteria, while two others independently performed data extraction.

### 2.3 Data extraction

Data were manually extracted from the full-text articles, including the first author, year of publication, sample size for human studies or PCOS induction method for animal studies, microbiota analysis techniques, alpha and beta diversity, and microbiota composition to report changes in gut microbiota composition, noting that we extracted the data of every available bacterial group using the lowest taxonomic level of each included study. For studies reporting treatment interventions, data included the first author, year of publication, sample size for human studies or PCOS induction method for animal studies, microbiota analysis techniques, alpha and beta diversity, microbiota composition, and changes in PCOS symptoms after treatment. For studies reporting metabolites, data extracted included the first author, year of publication, sample size for human studies or PCOS induction method for animal studies, microbiota analysis techniques, and metabolome shifts.

### 2.4 Risk of bias assessment

For human studies, the Methodological Index for Non-Randomized Studies (MINORS) scale was used to assess the risk of bias (Slim et al., 2003). The MINORS scale consists of 12 criteria, with a maximum score of 24, indicating low risk of bias. Higher scores reflect better methodological quality. For animal studies, we used the SYRCLE's Risk of Bias tool (Hooijmans et al., 2014), which contains 10 questions assessing potential bias. Each question was scored with “Yes” as 2 points, “No” as 0 points, and “Unclear” as 1 point. A higher overall score reflects a lower risk of bias.

### 2.5 Data synthesis

All reported outcomes were organized into tables describing different metrics and grid tables to display detailed differences in the gut microbiota from the phylum level down to species between PCOS case subjects and control groups. For visualizing microbiota changes, specific Excel conditional formatting functions were used: =ISNUMBER(SEARCH(D$2, $C3)) to color decreased gut microbiota data in red, and =ISNUMBER(SEARCH(D$2, $B3)) to color increased gut microbiota data in green. In these tables, column B listed all bacteria that increased, column C listed all bacteria that decreased, and row 2 contained the text needed for the conditional formatting functions to work.

The treatment tables were similarly formatted to highlight the impact of interventions on the gut microbiota. Data was extracted to show changes in microbiota composition and PCOS symptoms following treatment, with the same conditional formatting functions applied for visual clarity.

For the metabolites table, data were organized to show shifts in metabolome associated with PCOS. The same conditional formatting functions were utilized to highlight changes in metabolite levels, with specific formatting applied to visually distinguish shifts.

## 3 Results

### 3.1 Search results

**Figure 1:**
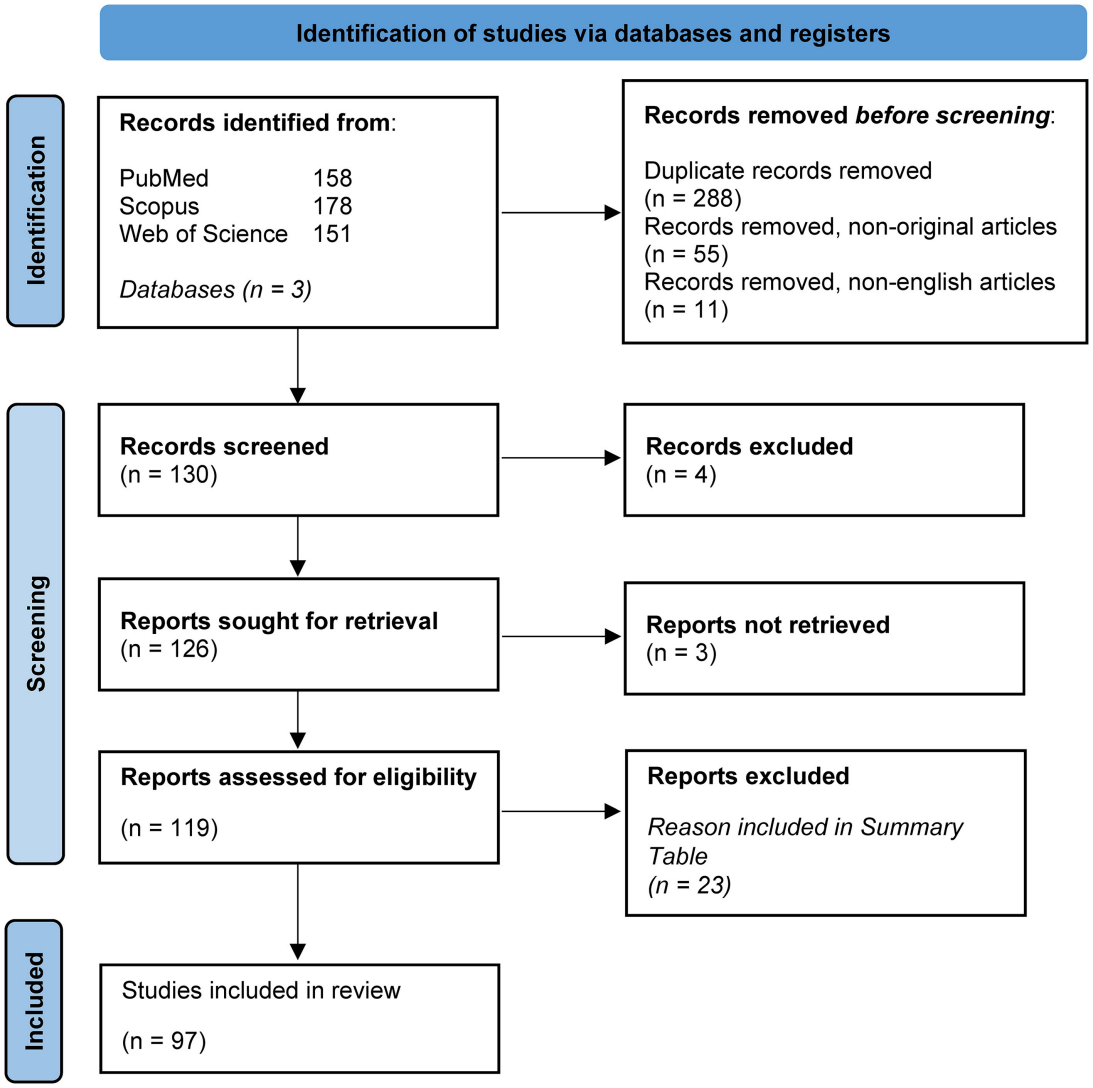
PRISMA flow diagram. This figure was adapted from PRISMA 2020 flow diagram for new systematic reviews which included searches of databases and registers only (source: Page et al., 2021). This work is licensed under CC BY 4.0. To view a copy of this license, visit https://creativecommons.org/licenses/by/4.0/.

We identified 288 studies after duplicate removal from three databases. After excluding non-original articles (reviews, systematic reviews, protocols, commentaries) and non-English articles, we screened 130 studies and removed 4 for focusing on the vaginal and salivary microbiota, which were out of scope. We attempted to retrieve 126 articles, but 3 were inaccessible, with no institutional or open access. In total, 119 full-text articles were assessed for eligibility, with 23 excluded for reasons detailed in Supplementary materials 2, 3. Ultimately, 97 studies were included in the review, with 4 discussing both human and animal models (Figure 1).

### 3.2 Study characteristics and assessment methods

#### 3.2.1 Human studies

Among the studies reviewed, they were sub-grouped into 3 categories: 31 compared the gut microbiota of PCOS patients with healthy controls, 9 investigated the effect of treatments on PCOS symptoms and the gut microbiota, comparing either with a placebo or pre-treatment baseline, and 18 focused on metabolomic shifts in gut microbiota-derived metabolites. In terms of sequencing methods, 26 studies used 16S rRNA sequencing, while 5 employed shotgun metagenomics for comparisons between PCOS and healthy controls. For studies evaluating treatment effects on the gut microbiota, 4 used 16S rRNA sequencing and 3 used shotgun metagenomics. Regarding metabolite analysis, 8 studies used KEGG pathway analysis, and 5 conducted untargeted metabolomics using methods such as HPLC and LC-MS. Risk of bias assessment score can be found in Supplementary material 2.

#### 3.2.2 Animal models

In animal models, 42 studies compared the gut microbiota before and after PCOS induction or with control groups, 34 evaluated treatment effects on PCOS symptoms and microbiota changes before and after treatment or compared with untreated animals, and 27 explored shifts in metabolites. In studies focusing on microbiota shifts, half of the studies used rats while the other half used mice. Regarding PCOS induction, 13 studies used DHEA, 18 used letrozole, 5 used DHT, and the remaining studies used other methods such as copper exposure and prenatal androgenization. All 42 studies employed 16S rRNA sequencing. For treatment studies, 33 used 16S rRNA sequencing, while 1 used shotgun metagenomics. In terms of animal type, 64% of studies used rats while 35% used mice, with 15 inducing PCOS using DHEA, 17 using letrozole, and 1 using DHT, while one study used testosterone propionate. For metabolite analysis, 17 studies focused on KEGG pathways, and 8 used untargeted metabolomics (HPLC, LC-MS). In these studies, 38.4% used mice, 61.5% used rats, with half inducing PCOS with DHEA and the other half with letrozole. Risk of bias assessment score can be found in Supplementary material 3.

### 3.3 Alteration in alpha and beta diversity

#### 3.3.1 Human studies (PCOS vs. healthy controls)

**Figure 2:**
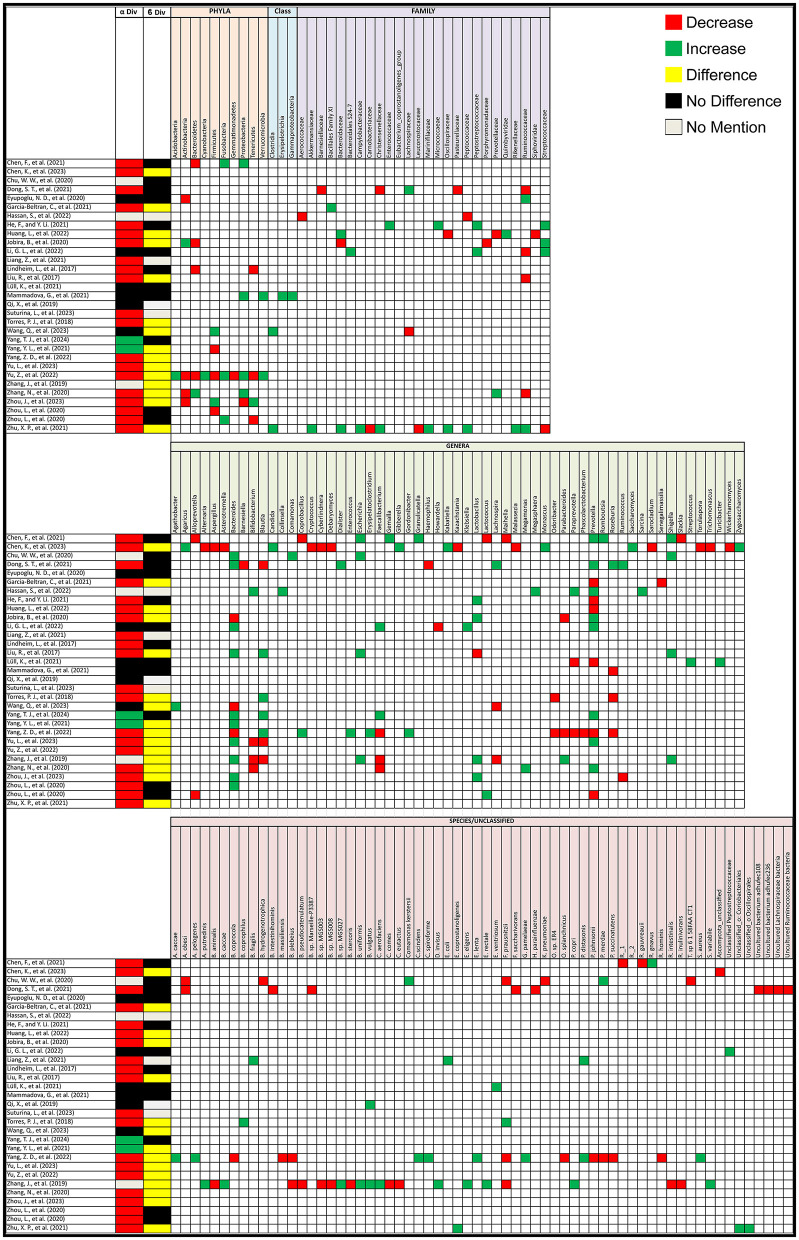
Gut microbiota alterations in PCOS patients. Grid table showing gut microbiome diversity and taxa alterations in PCOS Patients compared to healthy controls. Red colors show significant decreases in PCOS patients, green significant increases, black no significant change, yellow significant change.

Out of the 31 studies that compared alpha diversity between PCOS patients and healthy controls, 20 (64.5%) reported a decrease in alpha diversity in PCOS patients, 2 (6.45%) found an increase, and 6 (19%) observed no difference. Three studies (10%) did not assess alpha diversity. As for beta diversity, 15 (48%) studies reported significant changes between PCOS and healthy controls, 12 (39%) found no change, and 4 (13%) did not assess beta diversity (Figure 2).

#### 3.3.2 Animal studies (PCOS vs. healthy controls)

**Figure 3:**
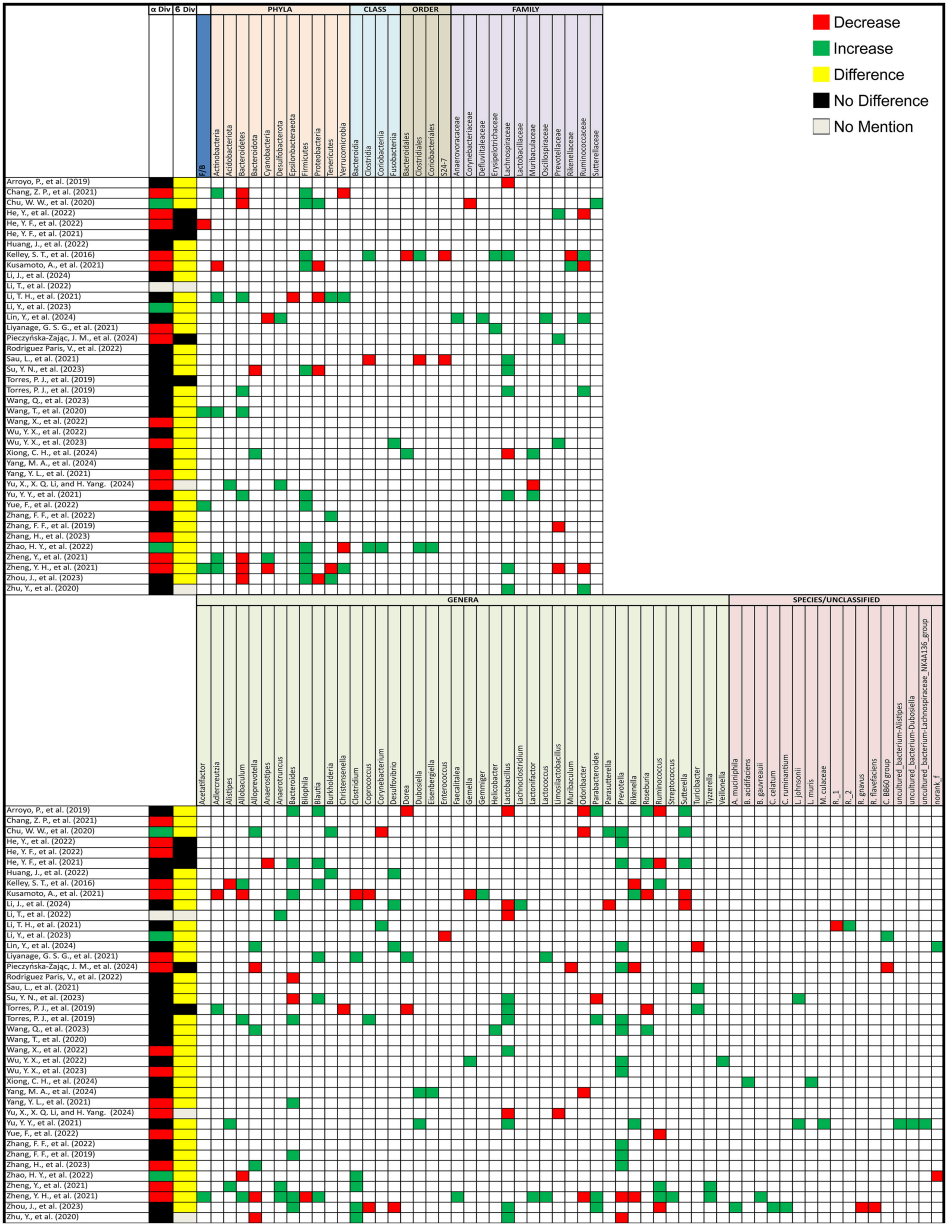
Gut microbiota alterations in PCOS animal models. Grid table showing gut microbiome diversity and taxa alterations in PCOS animal models compared to controls. Red colors show significant decreases in PCOS patients, green significant increases, black no significant change, yellow significant change, and gray no mention.

In animal models, out of 40 studies that compared alpha diversity between PCOS and healthy control groups, 15 (37.5%) reported a decrease, 3 (7.5%) observed an increase, and 21 (52.5%) found no difference. One study (2.5%) did not assess alpha diversity. For beta diversity, 32 (80%) studies reported significant changes between PCOS and healthy controls, while 5 (12.5%) found no change, and 3 (7.5%) did not assess beta diversity (Figure 3).

#### 3.3.3 Human studies (post-PCOS treatment)

**Figure 4:**
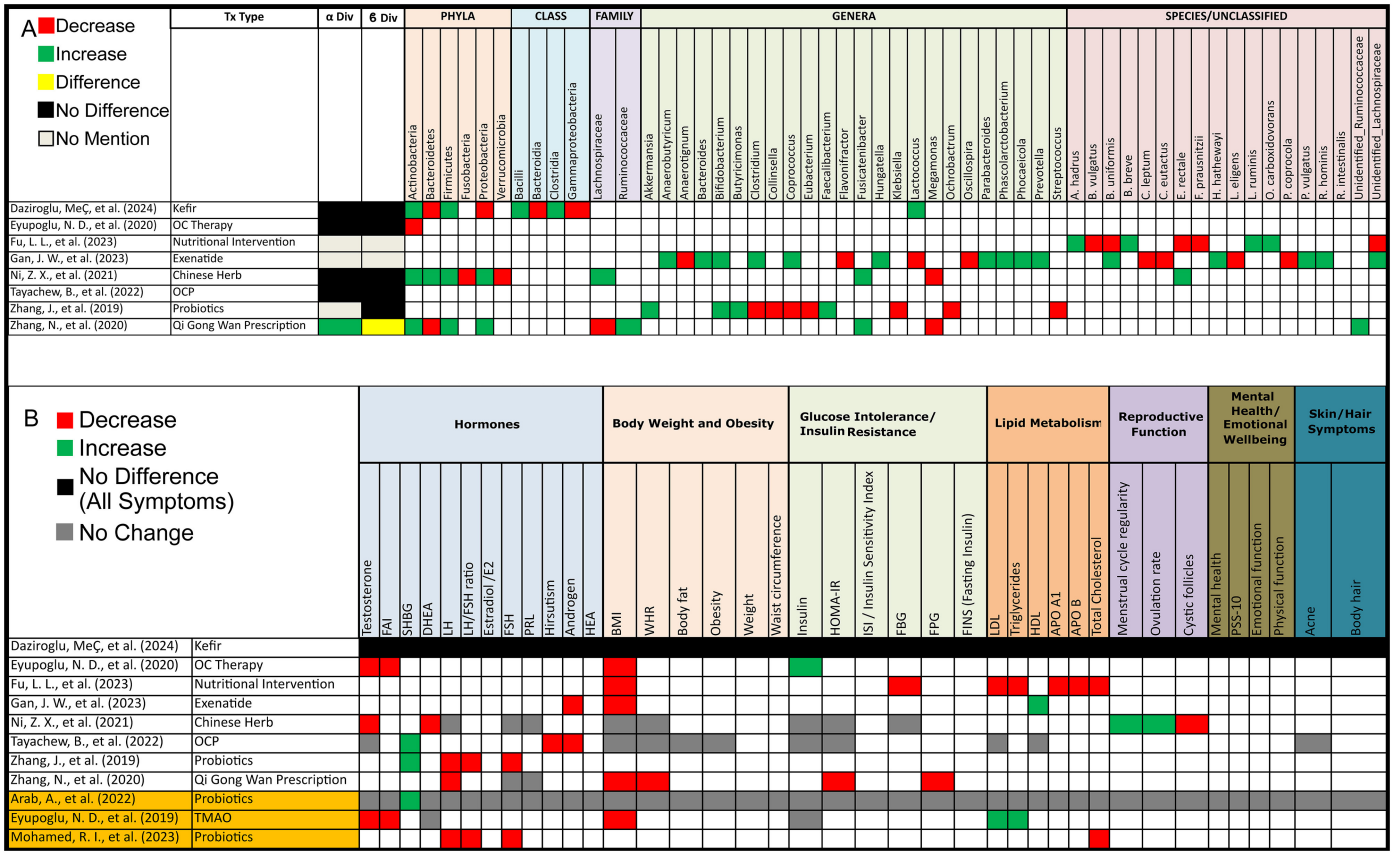
Gut microbiota and PCOS symptoms alterations post-treatment in PCOS patients. **(A)** Grid table showing gut microbiome diversity and taxa alterations in PCOS Patients Post-Treatment. Red colors show significant decreases post-treatment, green significant increases, black no significant change, yellow significant change, and gray no mention. **(B)** Grid table showing alterations in PCOS Symptoms Post-Treatment. Red colors show significant decreases post-treatment, green significant increases, black no significant change, yellow significant change, and gray no mention. Studies highlighted in orange are focused on pre/probiotic therapy.

Out of 8 studies that evaluated alpha diversity after PCOS treatment, 4 (50%) reported no difference, 1 (13%) found an increase, and 3 (37%) did not assess alpha diversity. In terms of beta diversity, 5 (63%) studies reported no change, 1 (13%) found a change, and 2 (25%) did not assess beta diversity (Figure 4A).

#### 3.3.4 Animal studies (post-PCOS treatment)

**Figure 5:**
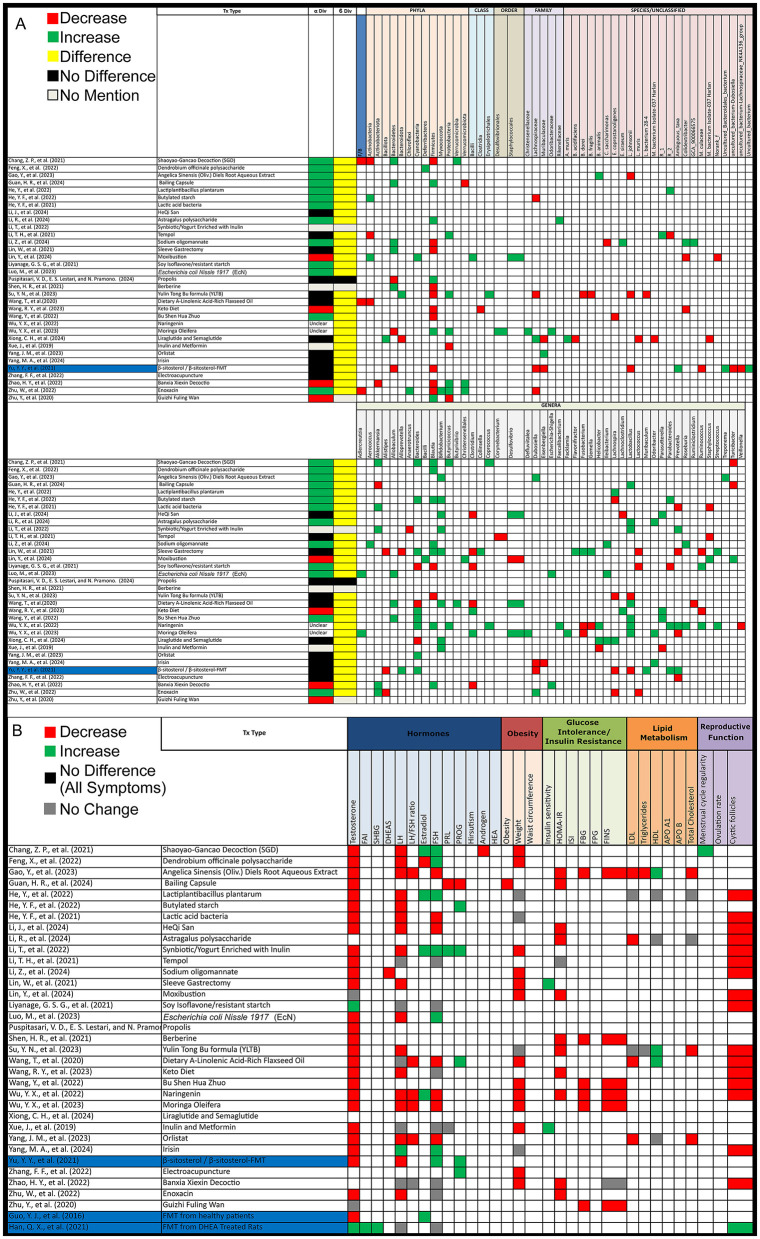
Gut microbiota and PCOS symptoms alterations post-treatment in PCOS animal models. **(A)** Grid table showing gut microbiome diversity and taxa alterations in PCOS models post-treatment. Red colors show significant decreases post-treatment, green significant increases, black no significant change, yellow significant change, and gray no mention. Highlighted in blue are studies working on FMT. **(B)** Grid table showing alterations in PCOS Symptoms Post-Treatment. Red colors show significant decreases post-treatment, green significant increases, black no significant change, yellow significant change, and gray no mention. Studies highlighted in blue are focused FMT.

Out of 33 animal studies assessing alpha diversity after PCOS treatment, 4 (12%) reported a decrease, 13 (39%) found an increase, 11 (33%) showed no change, 3 (9%) did not assess, and 2 (6%) had unclear results. For beta diversity, 29 (88%) studies showed significant changes following treatment, 1 (3%) reported no change, and 3 (9%) did not assess beta diversity (Figure 5A).

### 3.4 Alterations in gut microbiota composition

#### 3.4.1 Human studies

PCOS has been linked to significant changes in the gut microbiota. In human studies, particularly in the phyla *Actinobacteria, Bacteroidetes, Firmicutes, Fusobacteria, Proteobacteria*, and *Tenericutes*, and at the family and genus level, notable changes were seen in *Ruminococcaceae, Bacteroides, Bifidobacterium, Blautia, Escherichia-Shigella, Gemella, Lactobacillus, Lachnospira, Paraprevotella, Prevotella*, and *Roseburia*. The following alterations were observed in PCOS patients compared to healthy controls. At the phylum level, *Actinobacteria* and *Bacteroidetes* were both found to decrease in 4 studies but increasing in 1 study each, while *Firmicutes* showed a decrease in 3 studies and an increase in 2. *Fusobacteria* consistently increased in 3 studies, with no reports of a decrease. Similarly, *Proteobacteria* decreased in 3 studies but increased in 1, while *Tenericutes* showed a decrease in 3 studies and an increase in 1. At the family and genus level, notable changes were observed in several key microbial groups. *Ruminococcaceae* decreased in 4 studies and increased in 2. *Bacteroides* at the genus level saw a significant increase in 10 studies and a decrease in 3, while *Bifidobacterium* decreased in 3 studies and increased in 1. *Blautia* showed mixed results, increasing in 4 studies and decreasing in 3. *Escherichia* was reported to increase in all 4 studies that assessed it, and the *Escherichia-Shigella* group, including *Shigella*, also showed an increase in 4 studies. *Faecalibacterium* showed a decrease in 3 studies and an increase in 2, while *Lactobacillus* showcased an increase in 4 studies with a decrease in only one. *Lachnospira* and *Paraprevotella* were both reported to decrease in 2 studies and increase in 1. *Prevotella* exhibited variability, increasing in 8 studies but decreasing in 6, while *Roseburia* decreased in 3 studies and increased in 1. On the species level, *Faecalibacterium prausnitzii* showed a decrease in 3 studies but an increase in one. Interestingly, 8 studies reported changes in unclassified bacteria. Additional microorganisms reported in 2 or fewer studies, as well as the specific articles mentioned are listed in Figure 2, taxa grouped next to each other can be seen in Supplementary material 4.

Not only have bacterial gut microorganisms been linked to PCOS, but alterations in the gut mycobiome have also been observed. A study by Chen et al. (2023), highlighted that the mycobiota in PCOS patients can be significantly altered. In particular, *Saccharomyces, Candida, Zygosaccharomyces, Monascus, Gibberella, Agaricus*, and *Kabatiella* were found to increase in PCOS patients, while *Asterotremella, Ascomycota_unclassified, Trichomonascus, Cryptococcus, Cyberlindnera, Malassezia, Kazachstania, Aspergillus, Alternaria, Debaryomyces, Wickerhamomyces, Torulaspora*, and *Sarocladium* showed a decrease.

However, after treatment intervention we observed that as opposed to PCOS patients, *Actinobacteria* increased in 3 studies but decreased in one, *Bacteroidetes* decreased in 2 studies and increased in one, *Firmicutes* showed an increase in 3 studies, and *Proteobacteria* increased in 2 studies and decreased in 1, with 2 studies reporting variations in unidentified *Ruminococcaceae* and *Lachnospiraceae* (Figure 4A).

#### 3.4.2 Animal studies

For animal models, the *Firmicutes/Bacteroidetes (F/B) ratio* increased in 3 studies but decreased in 1. *Actinobacteria* increased in 5 studies but decreased in 1, *Bacteroidetes* decreased in 5 studies but increased in 4, and *Cyanobacteria* decreased in 2 studies but increased in 1. *Firmicutes* consistently increased in 11 studies, with no reports of a decrease. *Proteobacteria* increased in 4 studies but decreased in 1, and *Tenericutes* increased in 3 studies but decreased in 1. At a more specific taxonomic level, *Clostridia* and *Clostridiales* both increased in 2 studies but decreased in 1, *Lachnospiraceae* increased in 7 studies but decreased in 2, and *Ruminococcaceae* increased in 4 studies but decreased in 3. *Muribaculaceae* increased in 2 studies but decreased in 1, and *Prevotellaceae* increased in 3 studies but decreased in 2. For individual genera, *Alistipes* increased in 2 studies but decreased in 1, *Allobaculum* increased in 3 studies and decreased in 2, *Alloprevotella* increased in 4 studies but decreased in 3, and *Anaerotruncus* increased in 3 studies. *Bacteroides* showed a significant increase in 8 studies and a decrease in 2. *Blautia* increased in 6 studies, *Clostridium* increased in 6 studies but decreased in 1, and *Coprococcus* increased in 1 study but decreased in 2. *Lactobacillus* increased in 7 studies but decreased in 4, *Parabacteroides* varied with an increase in 3 studies but a decrease in 4, and *Prevotella* increased in 12 studies but decreased in 2. *Rikenella* increased in 2 studies but decreased in 3, *Roseburia* increased in 3 studies but decreased in 2, and *Ruminococcus* decreased in 4 studies but increased in 3. Finally, *Sutterella* increased in 3 studies but decreased in 2 while *Turicibacter* increased in 2 studies but decreased in one, additionally 7 studies showcased changes in uncultured bacterial groups. Additional microorganisms reported in 2 or fewer studies, as well as the specific articles mentioned are listed in Figure 3.

After treatment in animal models, the *Firmicutes/Bacteroidetes* (F/B) ratio decreased in 3 studies. *Actinobacteria* showed an increase and a decrease in 2 studies each, while *Bacteroidetes* increased in 5 studies and decreased in 3. *Firmicutes* decreased in 10 studies and increased in 5, and *Proteobacteria* increased in 5 studies and decreased in 2. *Verrucomicrobiota* increased in 2 studies and decreased in 1. At the family level, *Lachnospiraceae* decreased in 4 studies and increased in 1, while *Muribaculaceae* increased in 2 studies and decreased in 1. *Akkermensia* increased in 5 studies but decreased in 1. *Alistipes* decreased in 3 studies, while *Allobaculum* increased in 3 studies. *Bacteroides* increased in 8 studies and decreased in 3, and *Blautia* increased in 7 studies but decreased in 2. *Bifidobacterium* increased in 8 studies, while *Clostridium* decreased in 5 studies but increased in 2. Further alterations included *Desulfovibrio*, which increased in 3 studies and decreased in 1, and *Dubosiella*, which showed an increase and decrease in 2 studies each. *Fusobacterium* decreased in 2 studies and increased in 1, while *Helicobacter* increased in 2 studies and decreased in 1. *Ileibacterium* consistently increased in 3 studies, while *Lachnospira* decreased in 4 studies and increased in 2. *Lactobacillus* increased in 7 studies but decreased in 2, while *Lactococcus* decreased in 3 studies. Other notable changes include *Odoribacter* and *Parasutterella*, both of which increased in 3 studies with *Parasutterella* decreasing in 1, while Parabacteroides increased in 3 studies and decreased in 1. *Prevotella* decreased in 4 studies and increased in 3. *Ruminococcus* increased in 2 studies and decreased in 2, while *Staphylococcus* showed an increase in 2 studies and a decrease in 1 as opposed to *Turicibacter* with 2 decreases and an increase. Lastly, *Lactobacillus johnsonii* decreased in 3 studies, and *Colodextribacter* decreased in 2 studies and increased in 1. As for unclassified bacteria, 8 studies reported changes before and after treatment (Figure 5A).

### 3.5 Treatment effect on gut microbiota and PCOS symptom modulation

#### 3.5.1 Human studies

Treatment options for PCOS have been shown to induce shifts in both alpha and beta diversity, as well as changes in gut microbiota composition as mentioned previously. Interestingly, all treatment options demonstrated effects on at least one PCOS symptom. In contrast, one of the studies, found no effect on PCOS pathophysiology, though it did lead to microbiota shifts, Post-treatment (PT) increases were observed in *Firmicutes* (BT: 65.9%; PT: 69.2%) and *Actinobacteria* (BT: 1.8%; PT: 3.6%), with specific increases in *Clostridia* (BT: 63.2%; PT: 65.8%), *Bacilli* (BT: 0.8%; PT: 1.8%), and *Lactococcus* (PT: 2.8%). Decreases were seen in *Bacteroidetes* (BT: 28.0%; PT: 23.8%) and *Proteobacteria* (BT: 2.9%; PT: 1.8%), particularly in *Bacteroidia* (BT: 28.0%; PT: 23.6%) and *Gammaproteobacteria* (BT: 2.3%; PT: 1.0%), with an observed reduction in *Holdemania*.

For probiotic therapy, findings were more specific. One study revealed that after administering seven strains of beneficial bacteria, there was an increase in sex hormone binding globulin (SGHB), though no effect on other symptoms was noted. In another study, the administration of *Bifidobacterium lactis V9* led to improvements in several PCOS symptoms alongside gut microbiota shifts, although this was observed in only a subset of participants (9 out of 14; Figure 4). Lastly, prebiotic therapy using *Acacia senegal* (Arabic gum) resulted in reduced levels of LH, FSH, and total cholesterol (Figure 4).

#### 3.5.2 Animal studies

All treatment modalities resulted in alleviating at least one PCOS symptom, along with shifts in the gut microbiota as previously noted. A notable exception was treatment with soy isoflavones, which did not alleviate PCOS symptoms and even raised testosterone levels, although it reduced cystic follicles. This treatment led to an increase in *Ruminococcus* and decreases *in Blautia, Dorea, Lactococcus, Parabacteroides*, and genus *Clostridium*. Also, a study by Xiong et al. (2024), did not showcase treatment effect on PCOS symptoms and only stated an improvement in PCOS metabolism disorder, instead focusing on gut microbiota shifts.

Focusing on fecal microbiota transplantation (FMT), a study showed that FMT from healthy donors, as well as the administration of *Lactobacillus* as a probiotic, resulted in higher estradiol and estrone levels, along with lower testosterone and androstenedione levels, with FMT yielding more significant results. A second study, demonstrated that FMT from healthy donors in β-sitosterol-treated mice restored endometrial receptivity in PCOS-like mice. It also decreased FSH and progesterone levels while increasing LH and testosterone levels. Additionally, one study showed, that *Bacteroides vulgatus* could trigger PCOS while another demonstrated that FMT from DHEA-treated mice could induce PCOS symptoms in pseudo germ-free mice (Figure 5).

### 3.6 Gut microbiota related metabolites

#### 3.6.1 Human studies—untargeted metabolomics

In untargeted metabolomics of human studies, we observed significant increases in lipid production, particularly in metabolites such as 1-(4-benzylpiperazino)-2(pyridin-2-ylamino) propan-1-one, 4-(octyloxy)benzoic acid, acetylcarnitine, estrone sulfate, L-cystine, phosphatidylcholine (PC) (16:1/17:2), PC (22:6e/19:1), ethyl 2-cyano-3-(tetrahydro-3-thiophenylamino) acrylate, and sphingosine (SM) (d21:1/21:0). Changes in lipid metabolism were also noted, including increases in glycerophospholipid metabolites such as lysophosphatidylcholine (LPC) (18:2, 20:3, 18:1, P-16:0, 17:0, 15:0, 18:3, 20:4), PC, ganglioside GM3 (d18:0/16:0), and 1-linoleoylglycerophosphocholine. Additionally, unsaturated fatty acids like arachidonic acid (FC = 3.6), docosahexaenoic acid (FC = 4.8), and eicosatrienoic acid (FC = 5.2) showed increases, while certain lipids, such as LPC 17:2, LPC 18:2, and oxidized phospholipids (OxPC), were reduced.

In terms of short-chain fatty acids (SCFAs), there is a decrease in SCFAs, but one study showed an increase in acetic acid (*p* < 0.05) and propionic acid (*p* < 0.05).

Amino acid metabolism demonstrated elevated synthesis of valine in 2 out of 3 studies reporting it, leucine in 2 out of 4 with decreases observed in methionine, glutamine, arginine, phenylalanine, and tyrosine.

Other notable findings included changes in bile acids, such as a decrease in taurocholic acid and glycodeoxycholic acid (GDCA). Along with alterations in citric acid, choline with two decreases out of 3, glycerophosphocholine/phosphocholine, noting that glycerophosphocholine increased in one study and decreased in another, and antioxidants like porphyrin with 1 out of 2 decreases, and bilirubin with 2 reported decreases.

After PCOS treatment, there was a decrease in taurocholic acid and xanthine, as well as amino acids including L-threonine, L-tyrosine, L-serine, L-glycine, L-proline, and free L-carnitine. Conversely, there was an increase in several lipids, including palmitic acid, stearic acid, sphingosine 1-phosphate, palmitoleic acid, eicosenoic acid, erucic acid, tetracosanoic acid, and behenic acid.

**Figure 6:**
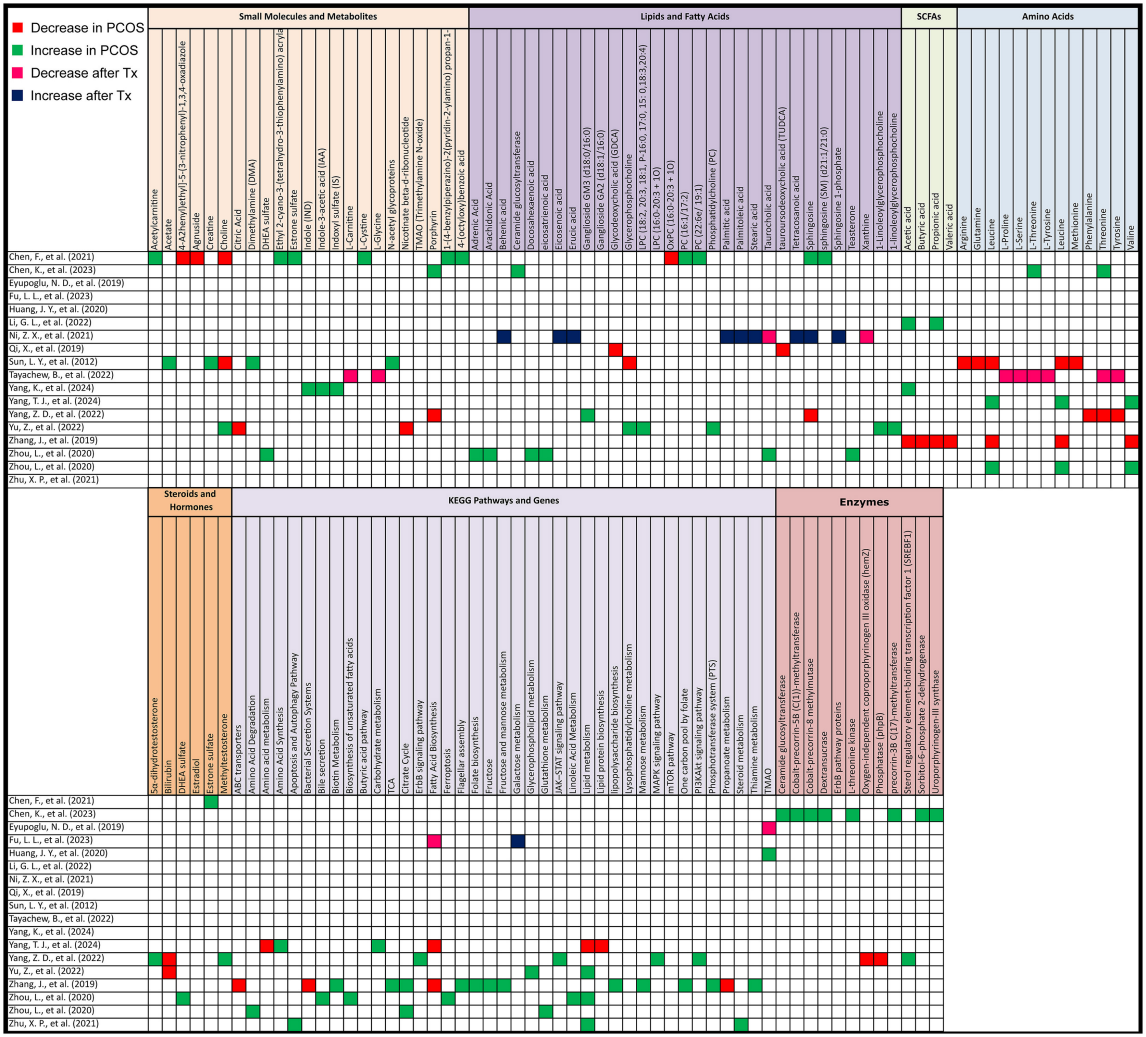
Gut metabolome alterations in PCOS patients. Grid table showing gut microbiome derived metabolites alterations in PCOS Patients compared to healthy controls, as well as post-treatment. Red colors show significant decreases in PCOS patients, green significant increases, Pink significant decrease post-treatment and blue significant increase post-treatment. If study has both PCOS patients and post-treatment results, only post-treatment is highlighted.

More specifically, in one of the studies, the microbial metabolite trimethylamine N-oxide (TMAO) increased in PCOS patients but decreased after oral contraceptive (OC) therapy (Figure 6).

#### 3.6.2 Human studies—KEGG pathway

In comparing PCOS to control groups, KEGG pathway analysis revealed significant differences. Lipid metabolism, particularly lipid biosynthesis, was elevated in three studies but decreased in one. Key pathways associated with energy metabolism were lower in PCOS, including the citric acid cycle (with mixed results) and the intermediate product nicotinate beta-d-ribonucleotide in the nicotinate and niacinamide metabolic pathway as mentioned in a study by Yu et al. (2022). Additionally, valine, leucine, and isoleucine biosynthesis, along with propanoate metabolism, ABC transporters, and bacterial secretion systems, were decreased in PCOS.

On the other hand, carbohydrate metabolism, certain amino acid synthesis (including valine, leucine, and isoleucine), and signaling pathways such as mTOR, ErbB, JAK–STAT, PI3K–Akt, and MAPK were increased. Sterol regulatory element-binding transcription factor 1, involved in insulin resistance and insulin signaling, was elevated. Other pathways that showed increases in PCOS were fructose and mannose metabolism, the citrate cycle, flagellar assembly, bacterial chemotaxis, cationic antimicrobial peptide resistance, lipopolysaccharide biosynthesis, phosphotransferase systems, biotin metabolism, folate biosynthesis, thiamine metabolism, and the one-carbon pool by folate. Lipid metabolism pathways, including biosynthesis of unsaturated fatty acids, linoleic acid metabolism, bile secretion, and ferroptosis, also showed increases in PCOS. Further differences included glutathione metabolism, steroid metabolism, and pathways involved in apoptosis and autophagy (Figure 6).

After treatment, fatty acid biosynthesis decreased in one study, while galactose metabolism was higher in another.

#### 3.6.3 Animal studies—untargeted metabolomics

In animal models, untargeted metabolomics after PCOS induction revealed several key metabolite changes. Lower levels were observed for 2-hydroxystearic acid, 3β-hydroxy-5-cholenoic acid, mesalazine, probucol, peptide YY (PYY), and secondary bile acids (12-ketoLCA, deoxycholic acid and tauroursodeoxycholic acid (TUDCA). There was also a reduction in interleukin-22 mRNA and short-chain fatty acids such as acetic acid, propionic acid, butyric acid, and pentanoic acid. Additionally, prostaglandin (i-19:0/LTE4), 3,4-dihydroxyphenylvaleric acid, and 8-iso-PGA1 were lower in abundance.

On the other hand, higher levels were detected for 1-octan-3yl primeveroside, acetamidobutanoic acid, 4-hydroxybenzaldehyde, tricin, argininosuccinic acid, betamethasone, daidzein, liquiritin, 2-(phenylethenyl)-1,3-dioxolane, androsta-1,4,6-triene-3,17-dione, and methallenestril. As for amino acids, Alanine was increased.

After treatment for PCOS, untargeted metabolomics revealed several metabolite shifts. Lower levels were observed in one study each for propionate (one study out of 3), acetic acid (one out of 2), and butyric acid (one out of 5). There was also a decrease in unsaturated fatty acids (UFA [CH = CH]) and glucose, alongside reductions in key metabolites such as testosterone, androstenedione, 17α-hydroxyprogesterone, adenosine monophosphate, 7α-hydroxytestosterone, tetrahydrocortisone, pregnenolone, etiocholanolone, and uridine diphosphate N-acetylglucosamine. Methallenestril and PS (22:5 [4Z, 7Z, 10Z, 13Z, 16Z]/LTE4) were also lowered.

**Figure 7:**
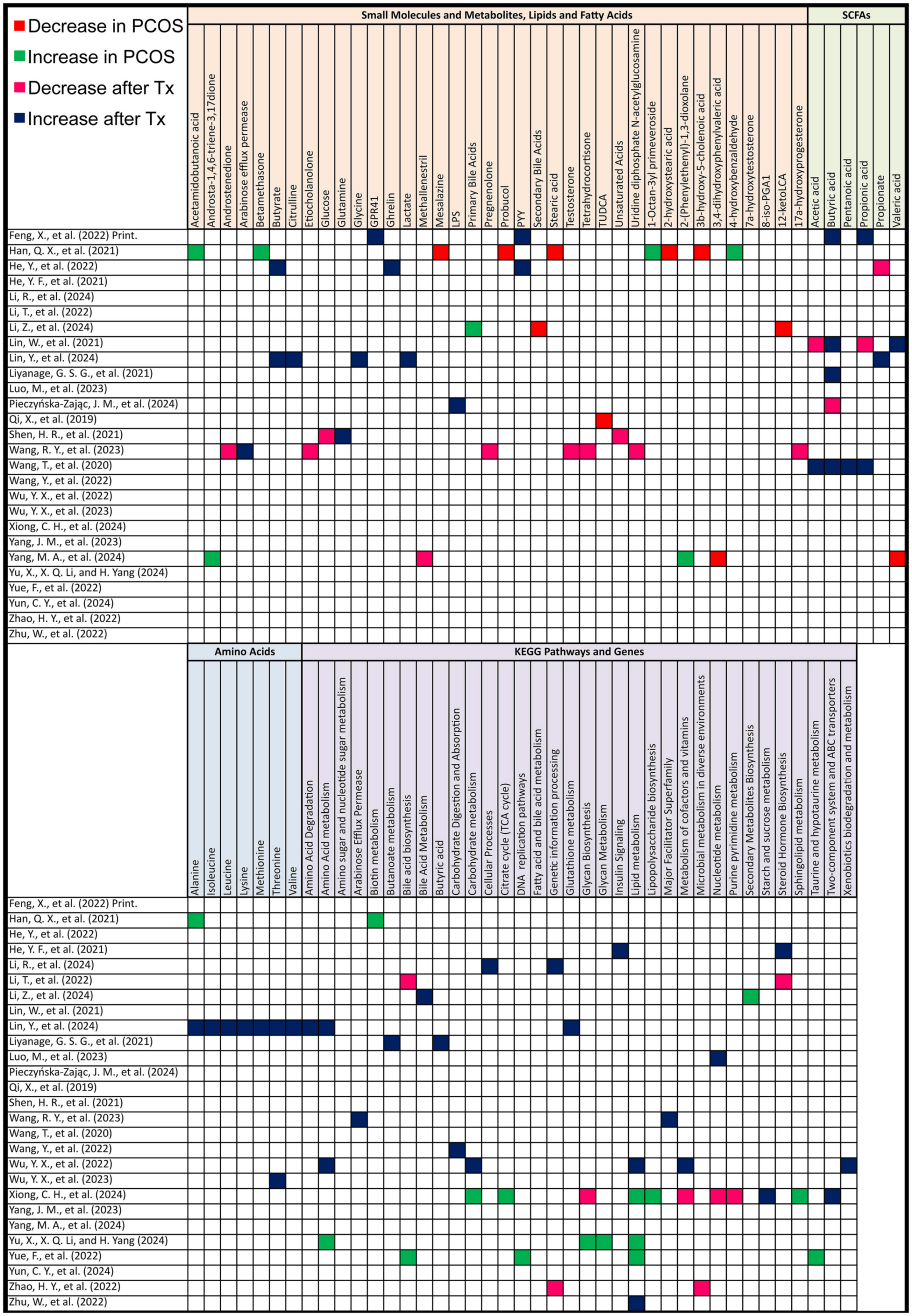
Gut metabolome alterations in PCOS animal models. Grid table showing gut microbiome derived metabolites alterations in PCOS models compared to controls, as well as post-treatment. Red colors show significant decreases in PCOS models, green significant increases, Pink significant decrease post-treatment and blue significant increase post-treatment. If study has both PCOS models and post-treatment results, only post-treatment is highlighted.

Conversely, higher levels were noted for butyric acid in 4 other studies, propionate in 2 others, and acetic acid in one. Propionic acid, serum PYY, colonic GPR41, and ghrelin were also found to increase. An increase in butanoate metabolism, butyric acid concentrations in a third study measuring butyric acid was observed. And plasma lipopolysaccharide (LPS) also showcased an elevation. Additionally, levels of glutamine, acetic acid, propionic acid, and pentanoic acid were elevated in another study measuring these SCFAs (Figure 7).

As for amino acids, Alanine, Isoleucine, Lysine, Methionine, Threonine, Valine were all elevated after treatment, as shown in the study by Lin et al. (2024).

#### 3.6.4 Animal studies—KEGG pathways

After PCOS induction, KEGG pathway analysis revealed significant upregulation in several pathways. These included biosynthesis of secondary metabolites and primary bile acids. Glycan biosynthesis and metabolism, global overview maps, carbohydrate metabolism, metabolism of cofactors and vitamins, lipopolysaccharide biosynthesis, glyoxylate, and lipid metabolism were also elevated. Additional increases were noted in amino acid metabolism, microbial metabolism in diverse environments, taurine and hypotaurine metabolism, bacterial systems, and DNA replication pathways.

After treatment, there was a decrease in bile acid biosynthesis (including both primary and secondary bile acid biosynthesis), steroid hormone biosynthesis, and glycan biosynthesis. Nucleotide metabolism, such as purine and pyrimidine metabolism, along with genetic information processing and microbial metabolism in diverse environments, also showed reductions.

Conversely, treatment resulted in an increase in pathways associated with steroid hormone biosynthesis, insulin signaling, fatty acid and bile acid metabolism, and cellular processes related to environmental information processing. There was also upregulation in the metabolism of butyrate, isoleucine, valine, propionate, lactate, lysine, leucine, acetate, methionine, taurine, glycine, threonine, and alanine. Other pathways elevated post-treatment included butanoate metabolism, amino sugar and nucleotide sugar metabolism, and carbohydrate digestion and absorption. Additionally, pathways related to lipid metabolism, xenobiotics biodegradation, and porphyrin metabolism were upregulated, along with key cellular responses to nitrogen compounds and hormones. Finally, molecular functions such as protein kinase activity and phosphotransferase activity also showed increases after treatment (Figure 7).

## 4 Discussion

The current study offers a comprehensive analysis of the complex relationship between the gut microbiota and PCOS, incorporating a detailed review of both human and animal studies. This examination extends to the effects of various treatments on PCOS and gut microbiota composition, as well as shifts in gut microbial metabolites in terms of PCOS. By addressing these interconnected factors, the study provides a thorough understanding of the multifaceted links between PCOS and the gut microbiota, thereby encompassing all relevant aspects of this critical relationship.

Alpha diversity measures the variety and balance of species within a single sample, with lower diversity often linked to metabolic dysfunction, such as in PCOS (Thackray, 2019). Most human studies showed a decrease in alpha diversity among PCOS patients, though some reported increases or no differences. Despite these variations, the general trend leaned toward decreased diversity, which was not reversed by treatment in most cases, except one (Zhang et al., 2020). In contrast, animal models showed more stability in alpha diversity, with over half reporting no changes after PCOS induction, though treatment more often led to increases. Beta diversity, which compares differences between samples, showed varied results in human studies, with 40% reporting no significant changes. Meanwhile, over 80% of animal studies showed significant shifts in beta diversity, both before and after treatment. This highlights inconsistent patterns between human and animal studies, although alpha diversity tends to decrease in PCOS patients, while beta diversity shows more consistent changes in animal models. The varying results in human studies could be attributed to differences in patient diets, as most studies did not control or regulate diet prior to analysis, introducing variability in gut microbiota composition and diversity measures (Conlon and Bird, 2014). In contrast, the beta diversity results in animal models may be more reliable due to the controlled and consistent diets provided during experiments, which likely reduces confounding factors.

Furthermore, in examining gut microbial and metabolic shifts in PCOS, we observe notable differences between human and animal models. Specifically, while *Bacteroides* demonstrated an increasing trend in both humans and mice with PCOS, other taxa exhibited divergent patterns. For instance, *Actinobacteria* increased in animal models, in contrast to the observed decrease in human studies. Similarly, *Firmicutes* showed a consistent increase, with no reports of a decrease, and there were significant increases in *Lachnospiraceae* and *Clostridium*. *Blautia*, too, showed a consistent rise without contradictory results. This contrasts with human studies where the representative taxa often exhibited more varied results. This discordance in gut perturbation between human and animal studies suggests a critical gap in our understanding. This gap highlights the necessity for employing humanized mice—germ-free mice colonized with human gut microbiota—to achieve a more accurate and comprehensive understanding of PCOS and its associated gut microbiota alterations (Nguyen et al., 2015).

Moreover, findings for most taxa in human studies were contradictory, with some more consistently reaffirmed, hinting at potential microbial markers. At the phylum level, *Actinobacteria* generally showed a decreasing trend, except for a study by Jobira et al. (2020), which reported an increase in PCOS patients and controls with obesity—likely explaining the divergence. *Bacteroidetes* demonstrated a consistent decrease, except in a study focusing on PCOS with phlegm dampness, where an increase was noted (Zhang et al., 2020). In accordance, *Actinobacteria* and *Bacteroidetes* decreases in PCOS are linked to a decrease in SCFAs and impact lipid metabolism (Johnson et al., 2017; Ventura et al., 2007). SCFAs such as acetate, propionate, and butyrate play an important role in metabolism due to their anti-inflammatory, anticarcinogenic, and immunomodulatory effects (Silva et al., 2020). The reduction in circulating SCFAs, showcased in our results, may contribute significantly to the decrease in insulin sensitivity and the development of insulin resistance, a major clinical feature of PCOS (Stener-Victorin et al., 2024; Canfora et al., 2015). Of note, vitamin D deficiency, which has been implicated in autoimmune diseases (Murdaca et al., 2019), has also been shown to influence gut dysbiosis by altering microbial composition and reducing SCFA-producing bacteria. Given that PCOS patients frequently exhibit lower vitamin D levels (Mohan et al., 2023), its deficiency may further exacerbate gut microbial imbalances and contribute to metabolic and immune dysfunction (Murdaca et al., 2021). This adds another layer of complexity to the gut metabolite alterations observed in PCOS, potentially linking vitamin D status to shifts in SCFA production and immune modulation. At the genus level, in contrast, *Bacteroides* was predominantly increased in 10 studies, with only 3 studies reporting a decrease (Jobira et al., 2020; Wang et al., 2023; Yang et al., 2022). Notably, *Bacteroides vulgatus* has been implicated in disrupting ovarian function, insulin resistance, altered bile acid metabolism, reduced interleukin-22 secretion, and infertility in colonized mice, as demonstrated by Qi et al. (2019). This comes in accordance with gut metabolite reductions observed across studies showing significant decreases in GDCA and TUDCA levels in PCOS patients as well as animal models. IL-22 levels were also reduced, hinting that, interventions targeting the gut microbiota, bile acid metabolism, and IL-22 modulation may be valuable for PCOS treatment. Additionally, *Escherichia-Shigella* and *Lactobacillus* were found to increase in multiple studies, noting that both these species play critical roles in metabolomic markers, especially SCFAs and lipid metabolism (Baltazar-Díaz et al., 2022; Le Roy et al., 2015). Adding that some *Lactobacillus* species, like *Lactobacillus acidophilus*, have been shown to be correlated with obesity, a hallmark of PCOS (Million et al., 2012). At the species level, *Faecalibacterium prausnitzii*, a bacterium with anti-inflammatory properties (Parsaei et al., 2021) consistently showed a decrease, linking it to the chronic inflammatory state of PCOS (Aboeldalyl et al., 2021). Interestingly, a study by Yu et al. (2022), LPCs, which are associated with metabolic disorders like inflammatory diseases, were significantly increased in PCOS patients. Though LPCs have been linked to insulin resistance, they also have glucose-lowering effects, highlighting the complex role of LPCs in human health and suggesting an area that needs further research (Liu et al., 2020). An intriguing finding is the large number of unclassified microorganisms, as well as findings that highlight shifts in the gut mycobiome, which broadens the understanding of microbial dysbiosis in PCOS beyond bacteria, suggesting that both bacterial and fungal communities may play crucial roles in PCOS pathophysiology (Chen et al., 2023). Importantly, both human and animal studies identified a high number of unclassified microorganisms, underscoring the need for advanced culturing techniques to better characterize novel gut microbiota and enhance species classification (Diakite et al., 2020), as these species can play a crucial role in a variety of diseases, particularly PCOS (Afzaal et al., 2022; Yu et al., 2022). These conflicting findings indicate that further research, using standardized protocols and larger sample sizes, is necessary to better understand these potential microbial markers and their roles in PCOS pathophysiology.

As for treatment interventions aimed at alleviating PCOS symptoms in both animal models and humans, alterations in the gut microbiota and associated metabolites were often observed. *Actinobacteria* levels increased across several studies, with some studies reporting reversal of changes induced by PCOS, specifically an increase in microorganisms with anti-inflammatory properties, like *Akkermensia municiphila* (Isnard et al., 2020). Furthermore, the mediators of the gut-brain axis—such as ghrelin, serotonin, and PYY—play a role in appetite regulation and psychological wellbeing in women with PCOS (Saydam and Yildiz, 2016) and their levels were found to increase after treatment, with Ghrelin and PYY negatively correlated with *Escherichia-Shigella* (Liu et al., 2017). Moreover, metabolites can serve as both disease markers and therapeutic targets. Taking as an example a study by Eyupoglu et al. (2019), where TMAO was elevated in PCOS patients but decreased following OC therapy, leading the authors to suggest TMAO as a potential contributor to PCOS pathophysiology. Probiotic and prebiotic treatments, while effective in certain cases, showed variable outcomes across studies, with some reporting significant improvements (Ma et al., 2019) and others showing minimal to no effect (Arab et al., 2022). One limitation with probiotics lies in the inconsistent colonization and sustainability of the introduced strains in the host's gut environment, which reduces their long-term efficacy (Merenstein et al., 2023). In contrast, FMT demonstrated more prominent effects compared to probiotics, particularly in the study by Guo et al. (2016), where FMT had a more significant impact on alleviating PCOS symptoms than Lactobacillus administration. Interestingly, FMT from DHEA-treated mice has been shown to induce PCOS in germ-free mice, which suggests that FMT from PCOS patients could also induce PCOS in animal models (Han et al., 2021). This could serve as a valuable future approach for creating a humanized PCOS model to further investigate the disease's interactions with the gut microbiota. When considering the combined data from these studies, it becomes evident that while FMT holds potential, it also has notable drawbacks—such as its invasive nature and the risk of adverse effects—making it an extreme and potentially severe option for treating PCOS (Giles et al., 2019). Similarly, the inconsistent outcomes of probiotic treatments further suggest a need for more reliable solutions. This has led to the idea that targeted live bacteriotherapy, which combines the advantages of both FMT and probiotics, could offer a promising therapeutic approach for PCOS, delivering specific strains that target the gut dysbiosis associated with the condition while avoiding the risks and inconsistencies of current treatments (Cheng et al., 2022; van Leeuwen et al., 2023).

Although all included studies were screened for bias using standardized tools and achieved good quality scores, certain limitations persist due to the nature of microbiome studies. One key limitation is the variability in assessment methods, including differences in DNA extraction protocols, sequencing platforms, and bioinformatics pipelines, which could not be fully standardized across studies. Additionally, most studies lacked dietary standardization, a crucial factor influencing gut microbiota composition (Delaroque et al., 2022), which may have contributed to variability in findings. Small sample sizes in some studies further limit generalizability, and the predominance of specific patient populations restrict diversity, potentially affecting the applicability of results to broader populations.

Considering these limitations, future studies should prioritize improving standardization across methodologies. Building upon these findings could facilitate the translation of microbiome research into clinical applications, whereas identifying microbial markers may improve diagnostic accuracy and early detection of PCOS, while microbiota-targeted interventions could expand treatment options for a condition that still lacks a conventional, universally effective therapy.

## 5 Conclusion

In conclusion, this study highlights inconsistent patterns between human and animal studies regarding gut microbiota alterations in PCOS. While alpha diversity tends to decrease in PCOS patients, beta diversity shows more consistent changes in animal models. Notably, increases in *Bacteroides vulgatus, Escherichia-Shigella* and *Lactobacillus* were observed, though findings across other taxa remain inconsistent. These inconsistencies underline the need for standardized protocols and larger sample sizes, limiting bias. Moreover, PCOS-driven disruptions across different metabolic pathways—including short-chain fatty acids, bile acids, and the gut-brain axis— labels it as multifaceted disease.

Additionally, the identification of numerous unclassified microorganisms across both human and animal studies highlights the importance of advanced culturing techniques to characterize novel gut microbiota, as well as employing humanized mice, which may also provide more accurate insights into the disease and its microbiota-related changes.

Regarding treatments, while fecal microbiota transplantation has shown promise, its invasive nature and potential risks make it a challenging option for treating PCOS, and the inconsistent outcomes of probiotic treatments stresses the need for more reliable therapeutic approaches.

The limitations of this review include the variability in assessment methods across studies, which could not be standardized. Additional limitations of the evidence involve the lack of diet standardization in most studies, small sample sizes in some, and a lack of diversity among patient populations.

Future research should focus on comprehensive treatment strategies, namely live bacteriotherapy, that focus on modifying gut microbiota and restoring metabolic balance, which may offer more effective solutions to the complexities of the disease. By integrating microbiome research into clinical practice, these advancements hold the potential to refine both diagnostic and therapeutic approaches, ultimately improving patient outcome.

## Data Availability

The original contributions presented in the study are included in the article/Supplementary material, further inquiries can be directed to the corresponding author.

## References

[B1] Aboeldalyl S. James C. Seyam E. Ibrahim E. M. Shawki H. E. Amer S. . (2021). The role of chronic inflammation in polycystic ovarian syndrome-a systematic review and meta-analysis. Int. J. Mol. Sci. 22:2734. 10.3390/ijms22052734 33800490 PMC7962967

[B2] Afzaal M. Saeed F. Shah Y. A. Hussain M. Rabail R. Socol C. T. . (2022). Human gut microbiota in health and disease: unveiling the relationship. Front Microbiol. 13:999001. 10.3389/fmicb.2022.999001 36225386 PMC9549250

[B3] Arab A. Hossein-Boroujerdi M. Moini A. Sepidarkish M. Shirzad N. Karimi E. . (2022). Effects of probiotic supplementation on hormonal and clinical outcomes of women diagnosed with polycystic ovary syndrome: a double-blind, randomized, placebo-controlled clinical trial. J. Funct. Foods 96:105203. 10.1016/j.jff.2022.105203

[B4] Baltazar-Díaz T. A. González-Hernández L. A. Aldana-Ledesma J. M. Peña-Rodríguez M. Vega-Magaña A. N. Zepeda-Morales A. S. M. . (2022). Escherichia/shigella, SCFAs, and metabolic pathways-the triad that orchestrates intestinal dysbiosis in patients with decompensated alcoholic cirrhosis from Western Mexico. Microorganisms 10:1231. 10.3390/microorganisms10061231 35744749 PMC9229093

[B5] Canfora E. E. Jocken J. W. Blaak E. E. (2015). Short-chain fatty acids in control of body weight and insulin sensitivity. Nat. Rev. Endocrinol. 11, 577–591. 10.1038/nrendo.2015.128 26260141

[B6] Chen K. Geng H. Liu J. Ye C. (2023). Alteration in gut mycobiota of patients with polycystic ovary syndrome. Microbiol. Spectr. 11:e0236023. 10.1128/spectrum.02360-23 37702484 PMC10580825

[B7] Cheng A. G. Ho P-Y. Aranda-Díaz A. Jain S. Yu F. B. Meng X. . (2022). Design, construction, and *in vivo* augmentation of a complex gut microbiome. Cell 185, 3617–3619. 10.1016/j.cell.2022.08.003 36070752 PMC9691261

[B8] Conlon M. A. Bird A. R. (2014). The impact of diet and lifestyle on gut microbiota and human health. Nutrients 7, 17–44. 10.3390/nu7010017 25545101 PMC4303825

[B9] Daniel N. Lécuyer E. Chassaing B. (2021). Host/microbiota interactions in health and diseases—Time for mucosal microbiology! *Mucosal Immunol*. 14, 1006–1016. 10.1038/s41385-021-00383-w 33772148 PMC8379076

[B10] Delaroque C. Wu G. D. Compher C. Ni J. Albenberg L. Liu Q. . (2022). Diet standardization reduces intra-individual microbiome variation. Gut Microbes 14:2149047. 10.1080/19490976.2022.2149047 36426908 PMC9704386

[B11] Dennett C. C. Simon J. (2015). The role of polycystic ovary syndrome in reproductive and metabolic health: overview and approaches for treatment. Diabetes Spectr. 28, 116–120. 10.2337/diaspect.28.2.116 25987810 PMC4433074

[B12] Diakite A. Dubourg G. Dione N. Afouda P. Bellali S. Ngom I. I. . (2020). Optimization and standardization of the culturomics technique for human microbiome exploration. *Sci*. Rep. 10:9674. 10.1038/s41598-020-66738-8 32541790 PMC7295790

[B13] Eyupoglu N. D. Caliskan Guzelce E. Acikgoz A. Uyanik E. Bjørndal B. Berge R. K. . (2019). Circulating gut microbiota metabolite trimethylamine N-oxide and oral contraceptive use in polycystic ovary syndrome. Clin. Endocrinol. 91, 810–815. 10.1111/cen.14101 31556132

[B14] Fava F. Rizzetto L. Tuohy K. M. (2019). Gut microbiota and health: connecting actors across the metabolic system. Proc. Nutr. Soc. 78, 177–188. 10.1017/S0029665118002719 30561288

[B15] Gibson-Helm M. Teede H. Dunaif A. Dokras A. (2017). Delayed diagnosis and a lack of information associated with dissatisfaction in women with polycystic ovary syndrome. J. Clin. Endocrinol. Metab. 102, 604–612. 10.1210/jc.2016-2963 27906550 PMC6283441

[B16] Giles E. M. D'Adamo G. L. Forster S. C. (2019). The future of faecal transplants. Nat. Rev. Microbiol. 17:719. 10.1038/s41579-019-0271-9 31534208

[B17] Guo Y. Qi Y. Yang X. Zhao L. Wen S. Liu Y. . (2016). Association between polycystic ovary syndrome and gut microbiota. PLoS ONE 11:e0153196. 10.1371/journal.pone.0153196 27093642 PMC4836746

[B18] Han Q. Wang J. Li W. Chen Z. J. Du Y. (2021). Androgen-induced gut dysbiosis disrupts glucolipid metabolism and endocrinal functions in polycystic ovary syndrome. Microbiome 9:101. 10.1186/s40168-021-01046-5 33957990 PMC8103748

[B19] Hooijmans C. R. Rovers M. M. de Vries R. B. M. Leenaars M. Ritskes-Hoitinga M. Langendam M. W. . (2014). SYRCLE's risk of bias tool for animal studies. BMC Med. Res. Methodol. 14:43. 10.1186/1471-2288-14-43 24667063 PMC4230647

[B20] Hou K. Wu Z-X. Chen X-Y. Wang J-Q. Zhang D. Xiao C. . (2022). Microbiota in health and diseases. Signal. Transduct. Target Ther. 7:135. 10.1038/s41392-022-00974-4 35461318 PMC9034083

[B21] Isnard S. Lin J. Fombuena B. Ouyang J. Varin T. V. Richard C. . (2020). Repurposing metformin in nondiabetic people with HIV: influence on weight and gut microbiota. Open Forum Infect. Dis. 7:ofaa338. 10.1093/ofid/ofaa338 32964062 PMC7489545

[B22] Jobira B. Frank D. N. Pyle L. Silveira L. J. Kelsey M. M. Garcia-Reyes Y. . (2020). Obese Adolescents With PCOS Have Altered Biodiversity and Relative Abundance in Gastrointestinal Microbiota. J. Clin. Endocrinol. Metab. 105, e2134–e2144. 10.1210/clinem/dgz263 31970418 PMC7147870

[B23] Johnson E. L. Heaver S. L. Walters W. A. Ley R. E. (2017). Microbiome and metabolic disease: revisiting the bacterial phylum Bacteroidetes. J. Mol. Med. 95, 1–8. 10.1007/s00109-016-1492-2 27900395 PMC5187364

[B24] Le Roy C. I. Štšepetova J. Sepp E. Songisepp E. Claus S. P. Mikelsaar M. . (2015). New insights into the impact of Lactobacillus population on host-bacteria metabolic interplay. Oncotarget 6, 30545–30556. 10.18632/oncotarget.5906 26437083 PMC4741550

[B25] Lin Y. Zeng H. Lin J. Peng Y. Que X. Wang L. . (2024). Evaluating the therapeutic potential of moxibustion on polycystic ovary syndrome: a rat model study on gut microbiota and metabolite interaction. Front. Cell Infect. Microbiol. 14:1328741. 10.3389/fcimb.2024.1328741 38665877 PMC11043641

[B26] Liu P. Zhu W. Chen C. Yan B. Zhu L. Chen X. . (2020). The mechanisms of lysophosphatidylcholine in the development of diseases. Life Sci. 247:117443. 10.1016/j.lfs.2020.117443 32084434

[B27] Liu R. Zhang C. Shi Y. Zhang F. Li L. Wang X. . (2017). Dysbiosis of gut microbiota associated with clinical parameters in polycystic ovary syndrome. Front. Microbiol. 8:324. 10.3389/fmicb.2017.00324 28293234 PMC5328957

[B28] Ma C. Peng Q. Jiang S. Chen K. Fang Y. Zhang J. . (2019). Probiotic bifidobacterium lactis V9 regulates the intestinal microbiome in patients with polycystic ovary syndrome. Kexue Tongbao/Chinese Science Bulletin 64, 360–368. 10.1360/N972018-0058731020040

[B29] Merenstein D. Pot B. Leyer G. Ouwehand A. C. Preidis G. A. Elkins C. A. . (2023). Emerging issues in probiotic safety: 2023 perspectives. Gut Microbes 15:2185034. 10.1080/19490976.2023.2185034 36919522 PMC10026873

[B30] Million M. Angelakis E. Paul M. Armougom F. Leibovici L. Raoult D. . (2012). Comparative meta-analysis of the effect of Lactobacillus species on weight gain in humans and animals. Microb. Pathog. 53, 100–108. 10.1016/j.micpath.2012.05.007 22634320

[B31] Min Q. Geng H. Gao Q. Xu M. (2023). The association between gut microbiome and PCOS: evidence from meta-analysis and two-sample mendelian randomization. Front. Microbiol. 14:1203902. 10.3389/fmicb.2023.1203902 37555058 PMC10405626

[B32] Mohan A. Haider R. Fakhor H. Hina F. Kumar V. Jawed A. . (2023). Vitamin D and polycystic ovary syndrome (PCOS): a review. Ann. Med. Surg. 85, 3506–3511. 10.1097/MS9.0000000000000879 37427232 PMC10328709

[B33] Murdaca G. Gerosa A. Paladin F. Petrocchi L. Banchero S. Gangemi S. . (2021). Vitamin D and microbiota: is there a link with allergies? Int. J. Mol. Sci. 22:4288. 10.3390/ijms22084288 33924232 PMC8074777

[B34] Murdaca G. Tonacci A. Negrini S. Greco M. Borro M. Puppo F. . (2019). Emerging role of vitamin D in autoimmune diseases: an update on evidence and therapeutic implications. Autoimmun. Rev. 18:102350. 10.1016/j.autrev.2019.102350 31323357

[B35] Nguyen T. L. Vieira-Silva S. Liston A. Raes J. (2015). How informative is the mouse for human gut microbiota research? Dis. Model. Mech. 8, 1–16. 10.1242/dmm.017400 25561744 PMC4283646

[B36] Page M. J. McKenzie J. E. Bossuyt P. M. Boutron I. Hoffmann T. C. Mulrow C. D. . (2021). The PRISMA 2020 statement: an updated guideline for reporting systematic reviews. BMJ 372:n71. 10.1136/bmj.n71 33782057 PMC8005924

[B37] Parsaei M. Sarafraz N. Moaddab S. Y. Ebrahimzadeh Leylabadlo H. (2021). The importance of *Faecalibacterium prausnitzii* in human health and diseases. New Microbes New Infect. 43:100928. 10.1016/j.nmni.2021.100928 34430035 PMC8365382

[B38] Qi X. Yun C. Sun L. Xia J. Wu Q. Wang Y. . (2019). Gut microbiota-bile acid-interleukin-22 axis orchestrates polycystic ovary syndrome. Nat. Med. 25, 1225–1233. 10.1038/s41591-019-0509-0 31332392 PMC7376369

[B39] Salari N. Nankali A. Ghanbari A. Jafarpour S. Ghasemi H. Dokaneheifard S. . (2024). Global prevalence of polycystic ovary syndrome in women worldwide: a comprehensive systematic review and meta-analysis. Arch. Gynecol. Obstet. 310, 1303–1314. 10.1007/s00404-024-07607-x 38922413

[B40] Saydam B. O. Yildiz B. O. (2016). Gut-brain axis and metabolism in polycystic ovary syndrome. Curr. Pharm. Des. 22, 5572–5587. 10.2174/1381612822666160715143933 27426125

[B41] Silva Y. P. Bernardi A. Frozza R. L. (2020). The role of short-chain fatty acids from gut microbiota in gut-brain communication. Front. Endocrinol. 11:25. 10.3389/fendo.2020.00025 32082260 PMC7005631

[B42] Slim K. Nini E. Forestier D. Kwiatkowski F. Panis Y. Chipponi J. . (2003). Methodological index for non-randomized studies (minors): development and validation of a new instrument. ANZ J. Surg. 73, 712–716. 10.1046/j.1445-2197.2003.02748.x 12956787

[B43] Stener-Victorin E. Teede H. Norman R. J. Legro R. Goodarzi M. O. Dokras A. . (2024). Polycystic ovary syndrome. Nat. Rev. Dis. Primers. 10:27. 10.1038/s41572-024-00511-3 38637590

[B44] Teede H. J. Tay C. T. Laven J. Dokras A. Moran L. J. Piltonen T. T. . (2023). Recommendations from the 2023 international evidence-based guideline for the assessment and management of polycystic ovary syndrome†. Fertil. Steril. 120, 767–793. 10.1016/j.fertnstert.2023.07.025 37589624

[B45] Thackray V. G. (2019). Sex, microbes, and polycystic ovary syndrome. Trends Endocrinol. Metab. 30, 54–65. 10.1016/j.tem.2018.11.001 30503354 PMC6309599

[B46] The Lancet Regional Health-Europe (2022). Polycystic ovary syndrome: what more can be done for patients? Lancet Reg. Health Eur. 21:100524. 10.1016/j.lanepe.2022.100524 36406775 PMC9666923

[B47] van Leeuwen P. T. Brul S. Zhang J. Wortel M. T. (2023). Synthetic microbial communities (SynComs) of the human gut: design, assembly, and applications. FEMS Microbiol. Rev. 47:fuad012. 10.1093/femsre/fuad012 36931888 PMC10062696

[B48] Ventura M. Canchaya C. Tauch A. Chandra G. Fitzgerald G. F. Chater K. F. . (2007). Genomics of actinobacteria: tracing the evolutionary history of an ancient phylum. Microbiol. Mol. Biol. Rev. 71, 495–548. 10.1128/MMBR.00005-07 17804669 PMC2168647

[B49] Wang Q. Sun Y. Zhao A. Cai X. Yu A. Xu Q. . (2023). High dietary copper intake induces perturbations in the gut microbiota and affects host ovarian follicle development. Ecotoxicol. Environ. Saf. 255:114810. 10.1016/j.ecoenv.2023.114810 36948015

[B50] Xiong C. Wu J. Ma Y. Li N. Wang X. Li Y. . (2024). Effects of glucagon-like peptide-1 receptor agonists on gut microbiota in dehydroepiandrosterone-induced polycystic ovary syndrome mice: compared evaluation of liraglutide and semaglutide intervention. Diabetes Metab. Syndr. Obes. 17, 865–880. 10.2147/DMSO.S451129 38406269 PMC10894520

[B51] Yang Z. Fu H. Su H. Cai X. Wang Y. Hong Y. . (2022). Multi-omics analyses reveal the specific changes in gut metagenome and serum metabolome of patients with polycystic ovary syndrome. Front. Microbiol. 13:1017147. 10.3389/fmicb.2022.1017147 36338055 PMC9627625

[B52] Yu Z. Qin E. Cheng S. Yang H. Liu R. Xu T. . (2022). Gut microbiome in PCOS associates to serum metabolomics: a cross-sectional study. Sci. Rep. 12:22184. 10.1038/s41598-022-25041-4 36564416 PMC9789036

[B53] Zhang N. Li C. Guo Y. Wu H. C. (2020). Study on the intervention effect of Qi Gong Wan prescription on patients with phlegm-dampness syndrome of polycystic ovary syndrome based on intestinal flora. Evid. Based Complement. Alternat. Med. 2020:6389034. 10.1155/2020/6389034 33062017 PMC7545460

