# Supplementary material for: Systematic review of gut microbiota composition, metabolic alterations, and the effects of treatments on PCOS and gut microbiota across human and animal studies
Source: Front Microbiol. 2025 May 14;16:1549499. doi: 10.3389/fmicb.2025.1549499 (PMC12116390; doi:10.3389/fmicb.2025.1549499)
Supplement: Supplementary file 1 [file Supplementary_file_1.docx]

Supplementary Material

# Supplementary Data

**Queries:**

**Pubmed**

(((("Gastrointestinal microbiome"[Title/Abstract] OR "Gastrointestinal Microbiomes"[Title/Abstract] OR "Microbiome, Gastrointestinal"[Title/Abstract] OR "Gastrointestinal Microbial Community"[Title/Abstract] OR "Gastrointestinal Microbial Communities"[Title/Abstract] OR "Microbial Community, Gastrointestinal"[Title/Abstract] OR "Gut Microbiome"[Title/Abstract] OR "Gut Microbiomes"[Title/Abstract] OR "Microbiome, Gut"[Title/Abstract] OR "Gut Microflora"[Title/Abstract] OR "Microflora, Gut"[Title/Abstract] OR "Gastrointestinal Microflora"[Title/Abstract] OR "Microflora, Gastrointestinal"[Title/Abstract] OR "Gastrointestinal Flora"[Title/Abstract] OR "Flora, Gastrointestinal"[Title/Abstract] OR "Gut Flora"[Title/Abstract] OR "Flora, Gut"[Title/Abstract] OR "Gastrointestinal Microbiota"[Title/Abstract] OR "Gastrointestinal Microbiotas"[Title/Abstract] OR "Microbiota, Gastrointestinal"[Title/Abstract] OR "Gut Microbiota"[Title/Abstract] OR "Gut Microbiotas"[Title/Abstract] OR "Microbiota, Gut"[Title/Abstract] OR "Intestinal Microbiome"[Title/Abstract] OR "Intestinal Microbiomes"[Title/Abstract] OR "Microbiome, Intestinal"[Title/Abstract] OR "Intestinal Flora"[Title/Abstract] OR "Flora, Intestinal"[Title/Abstract] OR "Intestinal Microbiota"[Title/Abstract] OR "Intestinal Microbiotas"[Title/Abstract] OR "Microbiota, Intestinal"[Title/Abstract] OR "Intestinal Microflora"[Title/Abstract] OR "Microflora, Intestinal"[Title/Abstract] OR "Enteric Bacteria"[Title/Abstract] OR "Bacteria, Enteric"[Title/Abstract] OR "Gastric Microbiome"[Title/Abstract] OR "Gastric Microbiomes"[Title/Abstract] OR "Microbiome, Gastric"[Title/Abstract]) AND ("Polycystic Ovary Syndrome"[Title/Abstract] OR "Ovary Syndrome, Polycystic"[Title/Abstract] OR "Syndrome, Polycystic Ovary"[Title/Abstract] OR "Polycystic Ovarian Syndrome"[Title/Abstract] OR "Ovarian Syndrome, Polycystic"[Title/Abstract] OR "Polycystic Ovary Syndrome 1"[Title/Abstract] OR "Sclerocystic Ovarian Degeneration"[Title/Abstract] OR "Ovarian Degeneration, Sclerocystic"[Title/Abstract] OR "Sclerocystic Ovary Syndrome"[Title/Abstract] OR "Stein-Leventhal Syndrome"[Title/Abstract] OR "Stein Leventhal Syndrome"[Title/Abstract] OR "Syndrome, Stein-Leventhal"[Title/Abstract] OR "Sclerocystic Ovaries"[Title/Abstract] OR "Ovary, Sclerocystic"[Title/Abstract] OR "Sclerocystic Ovary"[Title/Abstract]))) NOT (review[Publication Type])) NOT (systematic review[Publication Type])

**Scopus and Web Of Science (WOS)**

(("Gastrointestinal microbiome" OR "Gastrointestinal Microbiomes" OR "Microbiome, Gastrointestinal" OR "Gastrointestinal Microbial Community" OR "Gastrointestinal Microbial Communities" OR "Microbial Community, Gastrointestinal" OR "Gut Microbiome" OR "Gut Microbiomes" OR "Microbiome, Gut" OR "Gut Microflora" OR "Microflora, Gut" OR "Gastrointestinal Microflora" OR "Microflora, Gastrointestinal" OR "Gastrointestinal Flora" OR "Flora, Gastrointestinal" OR "Gut Flora" OR "Flora, Gut" OR "Gastrointestinal Microbiota" OR "Gastrointestinal Microbiotas" OR "Microbiota, Gastrointestinal" OR "Gut Microbiota" OR "Gut Microbiotas" OR "Microbiota, Gut" OR "Intestinal Microbiome" OR "Intestinal Microbiomes" OR "Microbiome, Intestinal" OR "Intestinal Flora" OR "Flora, Intestinal" OR "Intestinal Microbiota" OR "Intestinal Microbiotas" OR "Microbiota, Intestinal" OR "Intestinal Microflora" OR "Microflora, Intestinal" OR "Enteric Bacteria" OR "Bacteria, Enteric" OR "Gastric Microbiome" OR "Gastric Microbiomes" OR "Microbiome, Gastric") AND ("Polycystic Ovary Syndrome" OR "Ovary Syndrome, Polycystic" OR "Syndrome, Polycystic Ovary" OR "Polycystic Ovarian Syndrome" OR "Ovarian Syndrome, Polycystic" OR "Polycystic Ovary Syndrome 1" OR "Sclerocystic Ovarian Degeneration" OR "Ovarian Degeneration, Sclerocystic" OR "Sclerocystic Ovary Syndrome" OR "Stein-Leventhal Syndrome" OR "Stein Leventhal Syndrome" OR "Syndrome, Stein-Leventhal" OR "Sclerocystic Ovaries" OR "Ovary, Sclerocystic" OR "Sclerocystic Ovary"))
